# Exploring dupilumab for asthma: from mechanistic insights to clinical outcomes, safety, and cost-effectiveness

**DOI:** 10.3389/fphar.2025.1631321

**Published:** 2025-08-06

**Authors:** Omar Z. Ameer, Ghaith K. Mansour, Raghad S. Al-Amoudi, Fahmi M. Abu‐Owaimer

**Affiliations:** ^1^ Department of Pharmaceutical Sciences, College of Pharmacy, Alfaisal University, Riyadh, Saudi Arabia; ^2^ Department of Physiology, College of Medicine, Alfaisal University, Riyadh, Saudi Arabia

**Keywords:** dupilumab, type 2 asthma, eosinophilia, IL-3/IL-4 blocker, biologics cost-effectiveness, inflammation

## Abstract

Type 2 (T2) inflammation underlies a substantial subset of moderate-to-severe asthma, contributing to persistent symptoms and frequent exacerbations. Dupilumab, a fully human immunoglobulin G subclass 4 (IgG4) monoclonal antibody, targets the interleukin-4 receptor alpha (IL-4Rα), thereby inhibiting both interleukin-4 (IL-4) and interleukin-13 (IL-13) signaling—which are key cytokines driving T2 inflammation. This review examines the formulation, pharmacological profile, clinical efficacy, safety, and cost-effectiveness of dupilumab in the treatment of asthma, with an emphasis on its role across T2-high and selected T2-low phenotypes. Dupilumab displays nonlinear pharmacokinetics, with approximately 61% bioavailability and a prolonged half-life that supports biweekly subcutaneous (SC) administration. Clinical trials have demonstrated significant reductions in asthma exacerbation rates, improvements in forced expiratory volume in one second (FEV_1_), and decreased oral corticosteroid (OCS) dependence in adults and children with moderate-to-severe asthma. The benefits are particularly robust in patients with elevated eosinophil counts or fractional exhaled nitric oxide (FeNO), although efficacy extends to some patients with T2-low profiles. Reported safety data show a favorable profile, with mild adverse events, such as injection-site reactions and nasopharyngitis, being the most common. Nonclinical studies using surrogate antibodies in animal models revealed no evidence of systemic toxicity, reproductive harm, or carcinogenicity, reinforcing the drug’s high therapeutic index. From a pharmacoeconomic perspective, dupilumab has been found to be cost-effective in Japan compared to other biologics such as benralizumab and mepolizumab for asthma treatment. It has also shown cost-effectiveness in countries such as South Korea and the United Kingdom, particularly among patients with frequent exacerbations or chronic OCS use. However, in settings such as the United States and Colombia, high drug acquisition costs limit its cost-effectiveness unless its use is restricted to high-risk populations. In summary, dupilumab provides a targeted and generally well-tolerated treatment option for severe asthma. It is approved as an add-on maintenance therapy for patients aged ≥6 years with moderate-to-severe asthma, particularly those with T2 inflammation. By maximizing clinical and economic benefits through precision-guided patient selection, dupilumab’s dual IL-4/IL-13 blockade makes it a versatile biologic—especially suited for T2-high and overlapping asthma phenotypes or patients with comorbidities such as nasal polyps, eosinophilic esophagitis, and atopic dermatitis.

## 1 Introduction and background on asthma and biologic therapies

Asthma is a chronic inflammatory airway disease characterized by episodic bronchoconstriction, airway hyper-responsiveness, and variable airflow obstruction. It affects an estimated 300 million people worldwide, and approximately 5%–10% of asthmatics have severe or refractory symptoms despite maximal standard therapy (Caminati et al., 2021). These symptomatic asthma patients experience frequent exacerbations requiring systemic corticosteroids and suffer greater morbidity (O'Byrne et al., 2019). Asthma pathology is heterogeneous; a major subtype is type 2 (T2) high asthma, which is driven by T helper 2 (Th2) lymphocytes and T2 cytokines (IL-4 and IL-13), and is often associated with eosinophilia and elevated immunoglobulin E (IgE) (Hammad and Lambrecht, 2021). In addition to Th2 cells, group 2 innate lymphoid cells (ILC2s) significantly contribute to T2 inflammation; they rapidly respond to epithelial-derived cytokines (IL-25, IL-33, and TSLP) and produce IL-5 and IL-13 (Wallrapp et al., 2018), promoting further eosinophilia and tissue responses in parallel to Th2-driven adaptive immunity. Differentially, ILC2s act as early, antigen-independent drivers of T2 inflammation, whereas Th2 cells mediate antigen-specific adaptive responses (Zhu, 2015). Traditional management includes inhaled corticosteroids and bronchodilators, but severe T2-high asthma may remain uncontrolled, prompting the development of targeted biologic therapies (Côté et al., 2020; Kyriakopoulos et al., 2021).

Biologic agents have revolutionized the treatment of severe asthma by targeting key immune mediators. Since the early 2000s, several monoclonal antibodies have been approved: omalizumab (anti-IgE) was first approved in 2003 for allergic asthma (D’Amato, 2003), followed by IL-5 pathway inhibitors (mepolizumab, reslizumab, and benralizumab) for eosinophilic asthma (Lombardi et al., 2024; Ridolo et al., 2020) and, more recently, dupilumab (anti-IL-4 receptor α) (Shirley, 2017) and tezepelumab (anti-TSLP) for broad T2 inflammatory conditions (Ridolo et al., 2020). These biologics have demonstrated substantial reductions in severe exacerbation rates and improvements in lung function in their respective target populations, enabling more personalized asthma therapy (Kolkhir et al., 2023). Dupilumab, the focus of this review, is a novel biologic agent that blocks IL-4 and IL-13 cytokine signaling, which is central to T2 inflammation in asthma. Clinically, dupilumab is a considerably promising treatment option for patients with moderate-to-severe asthma marked by eosinophilic inflammation, allergic sensitization, or dependence on oral corticosteroids (OCSs) (Deeks, 2019; Ricciardolo et al., 2021).

Clinically, dupilumab, through its anti-IL-4/IL-13 mechanism, is being explored as a treatment for additional T2 diseases, such as bullous pemphigoid, chronic spontaneous urticaria, pediatric asthma (ages 6–11), and various food and environmental allergies (Camela et al., 2024). Furthermore, dupilumab was approved by the FDA in 2019 for the treatment of chronic rhinosinusitis with nasal polyps (CRSwNP) in adults and in 2022 for treating eosinophilic esophagitis (EoE) in patients aged 12 years and older (Zhu, 2015; Lutzu et al., 2025).

This review provides a focused overview of dupilumab’s formulation, pharmacology, clinical efficacy, and safety while also comparing it with other biologics and examining its international cost-effectiveness. The development of biologic agents such as dupilumab has transformed the management of severe asthma by targeting specific immune pathways rather than relying on broad immunosuppression. Dupilumab selectively blocks IL-4 and IL-13 signaling without depleting lymphocytes or neutrophils, thereby preserving immune function and avoiding generalized immunosuppression. As a result, patients receiving dupilumab are not at an increased risk of systemic infections or malignancies, and routine infection-monitoring laboratory tests are not required during treatment.

An important clinical consideration is the safety of vaccinations during dupilumab therapy. Live attenuated vaccines should ideally be administered prior to treatment initiation due to theoretical concerns about immunomodulation. However, clinical studies to date have not demonstrated any major safety issues related to vaccine administration while on dupilumab (Camela et al., 2024).

A thorough understanding of the current therapeutic landscape for asthma, including the mechanisms, biologic targets, and clinical impact of available treatment options, is essential for advancing precision medicine. This approach aims to achieve better disease control, fewer exacerbations, and reduced reliance on systemic corticosteroids, ultimately improving both clinical outcomes and quality of life. Biologic therapies have significantly transformed asthma management, particularly for severe phenotypes that are unresponsive to conventional treatment. Dupilumab, through its targeted inhibition of IL-4 and IL-13 signaling, exemplifies this approach, demonstrating robust clinical efficacy and improved patient outcomes. Its dual cytokine blockade not only reduces exacerbations and enhances lung function but also minimizes dependence on systemic corticosteroids, thus improving the quality of life of patients previously limited by available therapeutic options.

## 2 Chemistry and formulation of dupilumab

Dupilumab is a fully human immunoglobulin G subclass 4 (IgG4) monoclonal antibody with kappa light chains produced by using recombinant DNA technology in Chinese hamster ovary cell culture (Janecki et al., 2025). The antibody has a molecular weight of approximately 147 kilodaltons (kDa) and consists of two heavy chains and two light chains, which is typical of the IgG4 structure (McCann et al., 2024) (Supplementary Figure S1). As an IgG4 subclass antibody, dupilumab is engineered to have reduced effector function, and IgG4 antibodies exhibit minimal complement activation and limited Fcγ receptor engagement. This feature is intentional, as dupilumab’s therapeutic effect is achieved through cytokine receptor blockade rather than cell depletion. Dupilumab binds to the IL-4Rα subunit, and through steric hindrance, it prevents IL-4 and IL-13 from engaging the receptor, thus inhibiting downstream signaling *via* STAT6 in the target cells (McCann et al., 2024; Topouzis et al., 2025).

From a development standpoint, dupilumab was created using Regeneron’s VelocImmune^®^ technology, which produces fully human antibodies by utilizing genetically engineered mice with humanized immune systems (Le et al., 2020). The IgG4 heavy chain has a stabilizing S228P mutation in the hinge region, a common modification for therapeutic IgG4 antibodies to prevent Fab-arm exchange. IgG4 can naturally swap halves with other IgG4 antibodies, and the S228P mutation reduces this, maintaining antibody integrity (Silva et al., 2015). Dupilumab’s complementarity-determining region sequences are optimized for high affinity to IL-4Rα, achieving sub-nanomolar binding affinity. The antibody is highly specific, and it does not bind to other cytokine receptors or unrelated antigens. Analytical characterization shows typical glycosylation for an IgG produced in the Chinese hamster ovary cells (predominantly G0 and G1 afucosylated complex glycans on the Fc region) (Senini et al., 2024; Srzentic et al., 2020). This glycosylation can affect Fc receptor interactions; however, since the IgG4 Fc region has low effector function, and dupilumab is afucosylated to a degree, it exhibits even lower antibody-dependent cellular cytotoxicity, which is desirable for its mechanism of action (Plichta et al., 2023).

Pharmaceutically, dupilumab was developed through a global collaboration agreement between Regeneron and Sanofi and is marketed under the brand name Dupixent^®^ (Shirley, 2017). The bulk antibody of dupilumab is manufactured and purified using standard monoclonal antibody production techniques. The final drug product is a sterile, preservative-free solution for subcutaneous (SC) injection (Seegräber et al., 2018), typically formulated at 150 mg/mL. It is clear to slightly opalescent and colorless to pale yellow, with a pH of 5.9 (Information NCfB, 2025). Commercial presentations include a pre-filled syringe or pen: 300 mg in 2 mL or 200 mg in 1.14 mL, respectively (Information NCfB, 2025) (Supplementary Figures S2A, B). Although the 2 mL volume is relatively high for SC use, it is generally well-tolerated and administered over 30s (Information NCfB, 2025). The latex-free device components are screened for extractables and leachables. Excipients include L-arginine hydrochloride, L-histidine, sodium chloride, polysorbate 80 (0.04% w/v), and water for injection (Information NCfB, 2025). Arginine HCl and histidine stabilize the antibody and buffer the formulation (Pelaia et al., 2022), with histidine maintaining a pH that supports both stability and injection comfort, contributing to low aggregation and fragmentation under stress conditions (Tan et al., 2019). Polysorbate 80 minimizes aggregation or adsorption, and osmolality is adjusted using chloride (Information NCfB, 2025). There are no preservatives or antimicrobial agents, so each syringe is single-use. The product must be refrigerated at 2–8 °C; however, it can be kept at room temperature for a short period (14 days) prior to use to ease administration for patients (Information NCfB, 2025). The stability data have shown that dupilumab remains stable throughout its shelf life with no significant degradation or aggregation when stored properly (Information NCfB, 2025). Generally, there are no known incompatibilities with common asthma medications. However, the dupilumab solution should not be mixed with other injectable drugs. The site of injection is usually the thigh or abdomen, and rotation is recommended to avoid lipoatrophy or irritation. Patients or caregivers can be trained to self-administer the SC injection (Information NCfB, 2025). Each batch of dupilumab undergoes rigorous quality control, including UV absorbance for concentration, size-exclusion HPLC for purity (>98% monomer), capillary electrophoresis for charge variants, IL-4Rα bioassay for binding potency, and assays for host-cell proteins and DNA (Rabe et al., 2018). All specifications meet the predefined criteria consistent with clinical trial materials, supporting the regulatory approval of its chemistry, manufacturing, and controls (Rabe et al., 2018). The formulation is designed for stability and low immunogenicity, reflecting advanced integration of biotechnology and pharmaceutical development.

## 3 Dupilumab’s mechanism of action and pharmacological effects

As a fully human monoclonal antibody of the IgG4 subclass, dupilumab targets the alpha subunit of the IL-4 receptor, IL-4Rα (Seegräber et al., 2018). By binding to IL-4Rα, dupilumab simultaneously blocks the signaling of both IL-4 and IL-13, two key Th2 cytokines that share this receptor component (McCann et al., 2024) (Supplementary Figure S3).

The cytokines IL-4 and IL-13 (Supplementary Figure S3A) play key roles in driving many features of T2 inflammation in asthma (Seegräber et al., 2018). IL-4 promotes B-cell class switching to IgE and the differentiation of Th2 cells, while IL-13 contributes to mucus hypersecretion, sub-epithelial fibrosis, airway hyper-responsiveness, and elevated fractional exhaled nitric oxide (FeNO) levels (Pelaia et al., 2022; Tan et al., 2019). Consequently, dupilumab curtails IgE production, eosinophil recruitment, and goblet cell mucus production, thereby addressing the core pathophysiologic processes in T2-high asthma (McCann et al., 2024). The dual IL-4/IL-13 blockade by dupilumab (Supplementary Figure S3B) halts the crucial inflammatory pathways, resulting in the downstream suppression of STAT6 activation and reduced expression of multiple T2 inflammation signature genes (Ricciardolo et al., 2021). Thus, pharmacological IL-4Rα antagonism by dupilumab is believed to reduce biomarkers of Th2 inflammation. This can be linked to the fact that in clinical studies utilizing dupilumab, results have shown reductions in blood and sputum eosinophils (after an initial transient increase in some patients), marked declines in FeNO levels, and, in some cases, lower total IgE over time with dampening of IgE production (Rabe et al., 2018). Pooled analyses across trials confirm that treatment with dupilumab lowers serum total IgE by 50%–77% from baseline. It also reduces blood eosinophil counts after an initial transient increase and decreases Th2-associated chemokines such as TARC (CCL17) and eotaxin-3 (Hamilton et al., 2021). These biomarker changes parallel clinical improvement in the treated patients. In asthma trials, reductions in FeNO and eosinophils are associated with improvements in lung function and exacerbation rates. Importantly, patients with higher baseline T2 biomarker levels were found to respond the best; for instance, those with elevated blood eosinophils and/or high FeNO have greater dupilumab efficacy with greater FEV_1_ gains and exacerbation reduction than those with low T2 markers (Bacharier et al., 2024). Consequently, from the clinical perspective, high baseline markers of Th2-driven diseases such as eosinophils or IgE can often predict a robust response to dupilumab (Hamilton et al., 2021). All of these changes in the biomarkers can be correlated with improved lung function and improved asthma control. Importantly, as an IgG4 antibody, dupilumab does not trigger complement or antibody-dependent cytotoxicity, focusing its action on cytokine neutralization without depleting immune cells (Reddel et al., 2022; Wenzel et al., 2013).

Notably, dupilumab’s mechanism of action overlaps with the inflammatory pathways involved in atopic comorbidities, such as atopic dermatitis, allergic rhinitis, and chronic rhinosinusitis with nasal polyps, all of which are associated with elevated levels of IL-4 and IL-13 cytokines (Pelaia et al., 2022). This overlap helps explain why asthma patients with these comorbid conditions often experience particular benefit from dupilumab treatment (Ricciardolo et al., 2021).

In summary, dupilumab works by blocking IL-4 and IL-13 signaling, thereby targeting upstream drivers of T2 inflammation. This mechanism enables it to modulate multiple allergic and eosinophilic processes that contribute to asthma severity (Pelaia et al., 2022; Grom et al., 2018).

## 4 Pharmacokinetic and pharmacodynamic behaviors of dupilumab

Dupilumab is administered *via* the SC route (Supplementary Figure S2) and exhibits nonlinear, target-mediated pharmacokinetics, a characteristic typical of monoclonal antibodies (Li et al., 2020). Following a single SC dose, the peak plasma concentration is achieved within a 1-week period (Gade et al., 2024). In order to achieve a steady-state trough level, i.e., the point at which the amount of dupilumab entering the body equals the amount being eliminated, resulting in stable trough concentrations across doses, repeated dosing every two weeks is implemented in the asthma regimen. This allows steady-state trough levels to be attained over a 16-week treatment period (Gade et al., 2024; Ghani et al., 2021). The absolute bioavailability of SC dupilumab is approximately 61%–64% across indications, with similar values observed in both asthma and atopic dermatitis (Gade et al., 2024; Ghani et al., 2021). Dupilumab’s volume of distribution is relatively limited to 4.8 ± 1.3 L, consistent with its confinement largely to the vascular and interstitial spaces (Gade et al., 2024). Metabolically, dupilumab is catabolized by proteolytic degradation into peptides and amino acids, such as endogenous IgG, with no specific organ metabolism (Diabetes NIo and Digestive, 2012). The elimination of dupilumab is characterized by a long terminal half-life on the order of 4–5 weeks. After steady-state dosing, it takes an average of 9 to 13 weeks for serum concentrations to become undetectable in adults and adolescents following discontinuation (Gade et al., 2024). Clearance is not meaningfully affected by age in patients ≥6 years old (Gade et al., 2024). Although not yet approved in kids with asthma under 6 years of age, dupilumab clearance is faster relative to adults in those age groups (Seegräber et al., 2018; Gade et al., 2024). Body weight influences drug exposure, as patients with higher weights tend to have lower trough concentrations, which is why pediatric dosing is weight-tiered (e.g., 100 mg vs. 200 mg) to achieve comparable exposure (Regeneron Pharmaceuticals IS, 2025). Furthermore, immunogenicity can also affect the concerned drug’s pharmacokinetics; a minority of patients may develop anti-drug antibodies (ADAs) that can reduce dupilumab levels (Regeneron Pharmaceuticals IS, 2025). In clinical trials, approximately 1%–4% of dupilumab-treated patients developed high-titer ADAs, and those cases sometimes had undetectable drug trough levels (Castro et al., 2018a). Generally, the incidence of neutralizing antibodies is low, and the majority of patients maintain adequate drug exposure.

Pharmacodynamically, dupilumab exerts dose-dependent suppression of T2 inflammation biomarkers. In asthma trials, dupilumab treatment led to significant reductions in FeNO, reflecting IL-13 blockade in the airway epithelium and lowered circulating inflammatory markers (Wenzel et al., 2013). Clinically, these molecular effects translate to improved lung function and fewer exacerbations, especially in patients with elevated baseline eosinophils or FeNO. For example, in a phase II study, dupilumab added to inhaled corticosteroid/long-acting beta agonist (ICS/LABA) therapy rapidly improved the critical lung function parameter FEV_1_ within 2 weeks and reduced Th2 biomarker levels compared to placebo (Wenzel et al., 2016).

In summary, dupilumab exhibits predictable absorption and prolonged persistence in circulation, allowing for convenient dosing every other week. Patients may not experience the full clinical benefits until steady-state trough levels are reached (16 weeks), even though early biomarker improvements (e.g., reduced eosinophils) may occur sooner. This underscores the importance of adhering to the dosing schedule to maintain therapeutic drug levels. Outcome-wise, the pharmacodynamic impact is evidenced by marked attenuation of key inflammatory pathways in asthma, which gives rise to dupilumab’s efficacy. Dupilumab’s mechanism of action distinctly establishes it as a potent treatment for severe asthma characterized by T2 inflammation. From the authors’ perspective, this precise dual cytokine blockade notably enhances its efficacy compared to broader immunosuppressive treatments, which frequently carry a higher risk of systemic adverse effects. Although other biologics provide selective inhibition of single cytokines, dupilumab’s simultaneous targeting of two pivotal inflammatory mediators uniquely addresses multiple critical disease pathways, thus rendering it especially advantageous for managing complex asthma phenotypes.

## 5 Dupilumab clinical efficacy: phase I–IV trials in adults and children

The potential of IL-4 and IL-13 blockade in asthma by dupilumab was first demonstrated in a phase IIa trial by Wenzel et al. (2013). This randomized study enrolled 104 adults with moderate-to-severe eosinophilic asthma (blood eosinophils ≥300 cells/μL or sputum eosinophils ≥3%) who were on medium-to-high doses of ICS and LABA. Patients received dupilumab 300 mg or placebo weekly for 12 weeks, with LABA stopped at week 4 and ICS tapered by week 9. The primary endpoint of asthma exacerbation after corticosteroid withdrawal occurred in 6% of dupilumab-treated patients *versus* 44% of patients on placebo, representing an 87% relative risk reduction. Dupilumab also significantly improved lung function (pre-bronchodilator FEV_1_) and asthma control scores, while reducing Th2 biomarkers, such as FeNO and IgE (Wenzel et al., 2013). This landmark study provided the first evidence that blocking IL-4Rα could maintain asthma stability even during corticosteroid tapering in a population predisposed to exacerbations. Adverse events in this short trial were mostly mild, with more injection-site reactions, nasopharyngitis, and headaches caused by dupilumab therapy (Wenzel et al., 2013) (Supplementary Figure S4).

Subsequently, a larger phase IIb dose-ranging trial (LIBERTY ASTHMA VENTURE Phase IIb, 2016) evaluated dupilumab as add-on therapy in a broader uncontrolled asthma population (Castro et al., 2018a). In this pivotal trial, 776 adults on high-dose ICS and LABA were randomized to receive either a placebo or one of four dupilumab regimens, receiving either 200 mg or 300 mg administered every 2 weeks or every 4 weeks for 24 weeks. The primary endpoint was the change in FEV_1_ at 12 weeks in the subgroup with blood eosinophils of 300 cells/μL or higher. Dupilumab produced dose-dependent improvements in FEV_1_; for example, in the eosinophilic subgroup at 12 weeks, FEV_1_ increased by a mean of 0.39 L–0.43 L with every 2-week dosing compared to 0.18 L with placebo (*p* < 0.01) (Wenzel et al., 2016). Additionally, dupilumab reduced severe exacerbations across all patients regardless of baseline eosinophil levels, with an overall 60%–70% reduction in exacerbation rate compared to that with placebo (Wenzel et al., 2016). These results indicate dupilumab’s efficacy even in patients with low T2 inflammation, although lung function gains were the greatest in those with high eosinophils. In terms of side effects, safety findings demonstrated a favorable profile, with no new major safety signals and comparable rates of adverse events between the dupilumab and placebo groups, aside from mild injection-site reactions (Wenzel et al., 2016).

The inference of this phase IIb trial is that dupilumab improves asthma outcomes irrespective of baseline eosinophil levels and maintains a favorable safety profile, supporting its use as an add-on therapy in uncontrolled asthma (Wenzel et al., 2016). The study also confirmed significant FEV_1_ improvement in patients with severe asthma and T2 inflammation. A 300-mg biweekly dosing regimen was established as the approved protocol, and blood eosinophil counts ≥300 cells/μL were identified as a potential predictive biomarker of response.

Subsequently, based on the success of the phase II trial, two phase III trials were conducted and published in 2018, firmly establishing dupilumab’s efficacy in severe asthma. The LIBERTY ASTHMA QUEST trial by Castro et al. (Castro et al., 2018a) enrolled 1,902 patients aged 12 years and older with moderate-to-severe asthma that was uncontrolled despite medium-to-high doses of ICS and up to two additional controller medications (Busse et al., 2018). Adolescent participants were randomized to receive either 200 mg or 300 mg of dupilumab every 2 weeks or a placebo over a 52-week treatment period. No minimum biomarker criteria were required for inclusion, allowing both T2-high and T2-low asthma patients to be represented. The dual primary endpoints were the annualized rate of severe exacerbations and the change in FEV_1_ at 12 weeks. Dupilumab significantly improved both endpoints, reducing annual severe exacerbations by approximately 46%–48% *versus* placebo and increasing FEV_1_ at 12 weeks by 0.13–0.2 L more than placebo (*p* < 0.001) (Castro et al., 2018b). Additionally, dupilumab improved patient-reported asthma control and quality of life. Post-hoc analysis showed that patients with higher baseline T2 inflammation markers, such as eosinophils or FeNO, experienced the greatest benefit, but even those without allergic biomarkers had a significant reduction in exacerbations (Castro et al., 2018b). For example, among patients with allergic asthma, which accounted for 56% of the cohort, dupilumab significantly reduced exacerbation rates, with similar effects observed in non-allergic patients. In both groups, outcomes improved progressively with higher baseline eosinophil counts and FeNO levels (Castro et al., 2018b). These findings confirmed dupilumab’s broad efficacy in uncontrolled asthma and highlighted that T2 biomarkers are predictors of the magnitude of the response but are not prerequisites for it. The net conclusion from this study is that dupilumab is generally well-tolerated, with an overall safety profile that is comparable to that of placebo. Minor adverse effects occurred, such as mild topical reactions at the therapy injection site (Castro et al., 2018b), Supplementary Figure S4. Notably, unlike in atopic dermatitis patients, the incidence of conjunctivitis was also low and did not differ from that of the placebo in those asthma patients (Regeneron Pharmaceuticals IS, 2025).

The second phase III trial of dupilumab, LIBERTY ASTHMA VENTURE by Rabe et al., addressed the critical need of patients with severe asthma requiring daily OCS (Rabe et al., 2018). In this 24-week trial, 210 adults with prednisone OCS-dependent asthma were randomized to receive 300 mg of dupilumab every 2 weeks or a placebo, alongside a standardized OCS-tapering protocol. By week 24, the dupilumab group showed a 70% mean reduction in OCS dose *versus* 42% with placebo (*p* < 0.001). Moreover, 48% of the dupilumab-treated patients achieved OCS independence, which is nearly double that of the placebo group (25%). Importantly, these reductions were achieved without any loss of asthma control. Dupilumab also reduced severe exacerbations by 59% (rate ratio 0.41) and improved FEV_1_ by 0.22 L compared to placebo. Adverse events included injection-site reactions (9% for dupilumab vs. 4% for placebo) and transient blood eosinophilia in 14% of dupilumab patients, which was not associated with worse outcomes (Rabe et al., 2018). The transient eosinophilia did not appear to predict worse clinical outcomes, but it reflects the complex immune modulation when IL-4/IL-13 is blocked, as the latter effect could potentially cause eosinophils to temporarily redistribute from the tissues to blood (Castro et al., 2018a). Overall, this reviewed trial demonstrated that dupilumab can significantly reduce or eliminate chronic OCS intake while improving asthma outcomes in severe asthmatic patients, a benefit that can transform patient medical care given the myriad side-effects of long-term OCS therapy (Khurana et al., 2020).

On another front, beyond the adult population, the use of dupilumab has also been investigated in children with asthma. The pivotal VOYAGE trial (Bacharier et al., 2021) evaluated dupilumab in 408 children aged 6–11 years with uncontrolled moderate-to-severe asthma (Bacharier et al., 2021). Participants received weight-based dupilumab dosing (100 mg or 200 mg every 2 weeks) or a placebo over 52 weeks (Bacharier et al., 2021). All had evidence of T2 inflammation (blood eosinophils ≥150 cells/μL or FeNO ≥20 ppb). Dupilumab significantly reduced the annualized severe exacerbation rate by 59% (0.31 vs. 0.75 per year; relative risk 0.41; *p* < 0.001) and improved lung function, with a mean increase in percent-predicted FEV_1_ of 10.5 *versus* 5.3 points at week 12 (treatment difference 5.2; *p* < 0.001) (Bacharier et al., 2021). Benefits were greater in children with baseline eosinophils ≥300 cells/μL. The safety profile was favorable, with adverse events and growth outcomes comparable to the placebo group. The data affirm the safety and efficacy of IL-4/IL-13 blockade across a broad age range, supporting dupilumab’s role as a targeted therapy for pediatric patients with T2-driven asthma (Bacharier et al., 2021).

Long-term extension studies, such as the open-label TRAVERSE trial, have followed patients for up to 2 years of treatment. These studies demonstrated sustained asthma control with no new safety concerns related to prolonged dupilumab use (Rhee et al., 2024). For example, over 96 weeks in the TRAVERSE study, patients maintained the FEV_1_ improvements gained during the parent trials, while severe exacerbation rates remained low, at an annualized rate of approximately 0.47 (Rhee et al., 2024; Wechsler et al., 2022).

Real-world evidence supports dupilumab’s clinical efficacy. In the US ADVANTAGE observational study, patients initiating dupilumab experienced a 69% reduction in severe exacerbations over 12 months compared to baseline (Pfeffer et al., 2024). A separate head-to-head real-world comparison with omalizumab showed a 44% lower annual exacerbation rate in dupilumab-treated patients (*p* < 0.0001) and a 28% reduction in OCS use (Bleecker et al., 2024). Although observational in nature, these findings suggest that dupilumab may provide superior outcomes in patients eligible for either therapy, particularly those with overlapping allergic or eosinophilic phenotypes.

Collectively, the clinical trial programs (phases I through IV), along with emerging real-world studies, establish dupilumab as an effective add-on therapy across the spectrum of moderate-to-severe asthma, significantly improving exacerbation rates, lung function, and corticosteroid dependence in both adults and children with T2-driven inflammatory asthma. Nevertheless, clinicians should carefully consider patient selection guided by T2 biomarkers to fully realize the therapeutic potential of dupilumab. This biomarker-driven approach underscores dupilumab’s suitability as a precision medicine tool that is particularly beneficial for optimizing clinical outcomes in targeted patient subgroups.

## 6 International guideline recommendations involving dupilumab

The incorporation of dupilumab into asthma management is reflected in recent international guidelines for severe asthma. The Global Initiative for Asthma (GINA) now includes dupilumab as a recommended add-on controller at the highest step (step 5) for patients with severe T2 asthma. This initiative, for instance, notes that for difficult-to-treat severe asthma characterized by eosinophilia or elevated FeNO, the addition of a biologic, such as dupilumab, alongside anti-IL-5 or anti-IgE options, should be considered after a scrutinized specialist assessment (Wechsler et al., 2022). For children aged 6–11 years, GINA recently added IL-5 antibody therapy as an option, joining omalizumab and dupilumab as step 5 biologics for the treatment of pediatric severe asthma (Venkatesan, 2023). Thus, dupilumab is recognized as an effective therapy for both adolescents/adults and children with severe T2 asthma that is not controlled by high-dose ICS/LABA.

In the United States, the National Asthma Education and Prevention Program (NAEPP) guidelines, through their focused updates, recognize biologics as add-on treatment options in patients with moderate-to-severe asthma (Cloutier et al., 2020), although specific recommendations continue to evolve. The National Institutes of Health (NIH)/NAEPP guidelines also emphasize the importance of using asthma phenotyping to guide the selection of biologic therapies for improved symptom control. For instance, they recommended anti-IgE therapy for allergic asthma, anti-IL-5 for eosinophilic asthma, and dupilumab as an option for eosinophilic or allergic asthma with frequent exacerbations, especially when maximal therapy has been reached (Cloutier et al., 2020). Dupilumab’s role is particularly noted in patients with both high eosinophils and allergic features or those with chronic corticosteroid dependence.

On another level, the joint reports of the American Thoracic Society (ATS) and the European Respiratory Society (ERS) task force on severe asthma issued formal recommendations on biologics, conditionally recommending dupilumab for specific severe asthma phenotypes. After a systematic evidence review, the ERS/ATS guidelines conditionally suggest dupilumab as an add-on therapy for adult patients with severe eosinophilic asthma and those with severe corticosteroid-dependent asthma, regardless of the eosinophil level (Khurana et al., 2020). Clinical trials have shown that dupilumab substantially reduces exacerbations and enables OCS tapering in these patient populations (Khurana et al., 2020). The ERS/ATS group also noted that dupilumab may benefit patients with comorbid severe nasal polyposis, an IL-4/IL-13-driven condition. This aligns with its approved indication for chronic rhinosinusitis with nasal polyps, providing an additional therapeutic application for the drug (Khurana et al., 2020). Building on the ERS endorsement, dupilumab is formally recommended for adults with chronic rhinosinusitis with nasal polyps that remain uncontrolled despite treatment with intranasal or systemic corticosteroids (Mullol et al., 2019). It was noted in clinical trials that adding SC dupilumab to intranasal corticosteroids yielded a least-squares mean nasal polyp score reduction of −1.9 *versus* −0.3 for placebo (*p* < 0.001) and significant Lund–Mackay CT improvements (Al-Ahmad et al., 2025). Moreover, the ERS strategy and various other national guidelines, such as the UK National Institute for Health and Care Excellence (NICE) and the Canadian Thoracic Society, have incorporated dupilumab for severe asthma with T2 inflammation that is not controlled by standard treatment (Venkatesan, 2023; Holguin et al., 2020; Wise, 2021). Ultimately, this established that biomarkers such as blood eosinophils ≥150 cells/μL, FeNO ≥20 ppb, or the need for maintenance OCSs (Pelaia et al., 2022) are used as criteria to identify patients likely to benefit from dupilumab. These criteria are reflected in its approved labeling (European Medicines Agency, 2017). Dupilumab is also approved as the first treatment for eosinophilic esophagitis in patients ≥12 years, with clinical phase III trials demonstrating significant reductions in esophageal eosinophil counts and dysphagia *versus* placebo (Dellon et al., 2022). Dupilumab is also approved as the first treatment for prurigo nodularis, with the U.S. FDA approval granted in 2022 for adults at a dose of 300 mg every 2 weeks following a 600-mg loading dose. Clinical phase III trials demonstrated significant reductions in pruritus scores and nodule counts compared to placebo (Yosip et al., 2023).

In summary, current international guidelines such as GINA, NIH, and ERS/ATS strongly position dupilumab as a high-tier treatment for severe asthma, particularly in eosinophilic and OCS-dependent cases and in pediatric patients with T2 inflammation after standard therapy optimization (Khurana et al., 2020). These endorsements not only reflect the growing clinical confidence in dupilumab but also underscore its established role in modern asthma management. However, despite its guideline-backed status affirming both efficacy and safety, translating these recommendations into real-world practice remains challenging. Patient heterogeneity and high treatment costs necessitate a nuanced, individualized approach. In the current view, dupilumab represents a paradigm shift in severe asthma care, but its success hinges on thoughtful patient selection and systemic support to ensure both clinical and economic sustainability.

## 7 Dupilumab’s safety profile, adverse events, and long-term outcomes

Dupilumab’s safety profile in asthma trials has been favorable and is generally similar to that of placebo, aside from injection-site reactions. In pooled analyses of asthma studies, the common adverse events of dupilumab with incidence ≥1% were injection-site erythema, nasopharyngitis, and oropharyngeal pain (Regeneron Pharmaceuticals IS, 2025) (Supplementary Figure S4). Injection-site reactions are usually mild in nature and characterized by transient redness or itching. Unlike in atopic dermatitis, where up to 10% of patients experience conjunctivitis or other ocular effects with dupilumab, the incidence of conjunctivitis in asthma patients is not a significant concern (Regeneron Pharmaceuticals IS, 2025). The product label specifically notes that in asthma trials, eye inflammation events occurred at a frequency similar to that observed with placebo (Regeneron Pharmaceuticals IS, 2025). Similarly, rates of headache, sinusitis, and upper respiratory tract infections were found to be comparable between dupilumab and placebo in controlled trials, and these are mostly related to the underlying disease or co-administered inhalation therapies (Regeneron Pharmaceuticals IS, 2025).

One notable laboratory finding with dupilumab is transient blood eosinophilia. In approximately 1%–4% of patients, usually those with very high baseline eosinophil levels, dupilumab treatment may lead to further increases in blood eosinophil counts that occasionally exceed 3,000 cells/μL. This can typically arise within the first 2–4 months of therapy (Castro et al., 2018a). These transient increases in blood eosinophils during dupilumab treatment could be a result of enhanced IL-5 signaling due to the relieved negative feedback from IL-4 and IL-13 blockade and reduced eosinophil migration into tissues caused by the downregulation of adhesion molecules and chemokines (Gandhi et al., 2016).

In the phase III QUEST trial, a small subset of patients treated with dupilumab developed transient eosinophil counts >3,000 cells/μL, but these normalized with continued treatment or after stopping, and the majority of these patients still benefited clinically (Rabe et al., 2018). In the corticosteroid-lessening VENTURE trial, 14% of dupilumab-treated patients experienced transient eosinophilia vs. 1% on placebo (Rabe et al., 2018). These cases were usually not associated with clinical symptoms; however, a few instances of hypereosinophilic syndrome or eosinophilic pneumonia have been reported in post-marketing; therefore, monitoring eosinophil counts during the initial months of dupilumab therapy can be prudent. Thus, if patients receiving dupilumab present with new respiratory symptoms and elevated eosinophil counts, clinicians are advised to evaluate for eosinophilic complications, although such occurrences are rare. Patients presenting with unexplained hypereosinophilia, particularly those with late-onset asthma or nasal polyps, should be carefully evaluated for eosinophilic granulomatosis with polyangiitis (EGPA). Failure to recognize and assess for EGPA in these patients may delay diagnosis, leading to the subsequent development of systemic vasculitis characterized by small-vessel inflammation and multi-organ involvement (Berti et al., 2020; Trivioli et al., 2020).

As a fully human antibody, dupilumab has a low immunogenic potential (Matsunaga et al., 2020). However, approximately 5–7% of patients in asthma trials developed ADAs, with the majority at low titer. A small fraction of <1% developed high-titer neutralizing antibodies, which could reduce dupilumab serum levels (Regeneron Pharmaceuticals IS, 2025), reducing drug effectiveness. In pivotal trials, efficacy did not differ markedly between ADA-positive and ADA-negative subjects, except in rare cases where neutralizing antibodies led to undetectable drug levels. Routine monitoring for ADAs is not performed in practice because of the low incidence and unclear clinical relevance.

Severe hypersensitivity reactions to dupilumab are uncommon. Anaphylaxis due to dupilumab was not observed in asthma trials (Castro et al., 2018a). During post-marketing, a few cases of generalized urticaria or serum sickness-like reactions have been described, so dupilumab is contraindicated in patients with known dupilumab allergy (Regeneron Pharmaceuticals IS, 2025). Any signs of severe hypersensitivity require immediate discontinuation of treatment and prompt medical evaluation to assess allergenicity. However, these events are very rare considering the large population of patients treated (Wechsler et al., 2022).

Unlike systemic immunosuppressants, dupilumab does not broadly suppress the immune system. In trials, the overall infection rates, including viral infections and bacterial pneumonia, were similar between the dupilumab and placebo groups (Regeneron Pharmaceuticals IS, 2025). Consequently, no routine laboratory monitoring is required during dupilumab therapy aside from optionally checking the eosinophil levels. The risk of opportunistic infections associated with this therapy is considered minimal. However, one consideration is parasitic infections—often helminth—in which IL-4 and IL-13 cytokines play important roles in host defense. Therefore, blocking these cytokines with dupilumab could theoretically increase susceptibility and vulnerability to such infestations (Pera et al., 2023). Clinical trial protocols excluded patients at high risk for helminthiasis and advised discontinuing dupilumab if a serious helminth infection occurs (Lopes and Desai, 2019). In practice, such cases have been exceedingly rare; a 2019 review found no reported helminth infections in asthmatics on dupilumab in trials (Castro et al., 2018a). Nonetheless, guidelines recommend screening for and treating parasitic infections in patients at risk, such as those who have traveled to endemic areas, before initiating dupilumab therapy (European Medicines Agency, 2017).

Long-term extension studies spanning up to 2–3 years have not revealed any new safety concerns. In an open-label extension for patients on continuous dupilumab for up to 3 years, the rates of adverse events were consistent with earlier trials and did not increase over time (Rhee et al., 2024). Specifically, there was no evidence of accumulating risk of serious infections, malignancies, or autoimmune diseases (European Medicines Agency, 2017). Asthma control remained durable: patients maintained improved lung function and low exacerbation rates through 96 weeks of treatment (Rhee et al., 2024). This suggests that the benefits of dupilumab are sustained with ongoing therapy, and tolerance does not wane over time. Additionally, some data indicate potential disease modification, as data analysis found that dupilumab-treated patients experienced a significantly slower decline in lung function over time than those receiving a placebo (Rhee et al., 2024), raising the possibility that early intervention could slow the progressive loss of lung function in severe asthma.

From a safety monitoring perspective, patients on dupilumab do not require routine laboratory monitoring, aside from optionally checking the eosinophil count. Unlike IL-5 antibodies, which dramatically lower eosinophil levels, dupilumab usually leaves eosinophils in the normal range or mildly elevated, as reiterated before. No specific organ toxicities (e.g., hepatic or renal) have been linked to dupilumab, and no dose adjustments are needed for comorbid conditions. With respect to vaccinations, since dupilumab modulates immune function, live vaccines are generally not recommended during treatment due to the theoretical risk of inadequate immune response (European Medicines Agency, 2017). Non-live vaccines, such as influenza or COVID-19 vaccines, are considered safe, and patients should stay up-to-date with them. Consolidating on this, only a few studies have shown that dupilumab does not significantly impair the antibody response to vaccines (Blauvelt et al., 2019).

In summary, dupilumab demonstrates a generally favorable safety profile within the asthma biologics landscape, making it a suitable option for long-term management in selected patients (Agache et al., 2020; Lipworth et al., 2025). The majority of adverse events, such as injection-site reactions and nasopharyngitis, are mild and manageable, while serious complications remain rare and typically transient. Notably, even concerns such as elevated eosinophil counts have not translated into significant long-term risks in clinical practice. When compared to traditional therapies such as OCS, which carry a substantial burden of systemic side effects, dupilumab offers a markedly safer alternative (Rabe et al., 2018). In contrast to concerns about long-term treatment risks, dupilumab’s strong safety profile combined with its sustained clinical efficacy positions it not merely as a viable option but as a preferred long-term strategy for managing severe asthma in appropriately selected patients.

## 8 Dupilumab’s comparative efficacy and safety versus other biologics

Dupilumab enters a therapeutic landscape alongside several other biologics for severe asthma, with therapeutic agents targeting different mechanistic components of the T2 inflammatory cascade in the asthma processes. For example, omalizumab targets IgE, mepolizumab/reslizumab target IL-5, benralizumab targets the IL-5 receptor, resulting in eosinophil depletion, and tezepelumab targets thymic stromal lymphopoietin (TSLP), an epithelial cytokine upstream of IL-4/5/13 (Venkatesan, 2023; Wise, 2021). Comparing the efficacy of these biologics is complex, as no head-to-head trials have been performed on asthma patients, and patient selection differs by phenotype. However, clinical trials of all these agents show broadly similar reductions in exacerbation frequency, as typified by 50% or more relative reduction relative to placebo in their target populations (Wenzel et al., 2016; Khurana et al., 2020). The key differences lie in which patients benefit most from each targeting therapy and specific secondary outcomes.

The IL-5 monoclonal antibodies, namely, mepolizumab and reslizumab, and the IL-5 receptor blocker, benralizumab, are highly effective in eosinophilic asthma (Venkatesan, 2023; Bondar and Carr, 2017; Menzella et al., 2016), especially when blood eosinophils are markedly elevated to ≥300 or 500 cells/μL or associated with frequent exacerbations. In such patients, anti-IL-5 biologics reduce exacerbation rates by 50% and improve FEV_1_ by 0.1–0.2 L (Castro et al., 2018a; Khurana et al., 2020). Dupilumab also shows strong efficacy in eosinophilic asthma, and many participants in dupilumab trials would have qualified for IL-5 therapy; in those with high eosinophil levels, exacerbation reductions were in the range of 60–70% (Khurana et al., 2020). An advantage of dupilumab is that it addresses additional pathways of IL-4/13 beyond eosinophil counts, potentially benefiting patients who, for example, have allergic comorbidities or high FeNO out of proportion to eosinophils. Trials suggest that dupilumab has significant efficacy even when eosinophils are low, provided other T2 markers such as FeNO are present (Castro et al., 2018b). In contrast, anti-IL-5 agents show minimal benefit in low-eosinophil populations.

Dupilumab can also improve nasal polyposis and dermatitis (Li et al., 2020), conditions that IL-5-targeting agents generally do not directly treat. Although mepolizumab may have some efficacy in treating nasal polyps, it is not currently approved for this indication. In terms of lung function, benralizumab tends to produce FEV_1_ gains of 0.25–0.3 L in patients with eosinophilic asthma that are comparable to dupilumab’s gains in similar subgroups (Castro et al., 2018b). Although no direct head-to-head comparisons exist, a network meta-analysis found no significant differences in exacerbation reduction between dupilumab, mepolizumab, and benralizumab when each was administered to appropriately selected phenotype groups, thus underscoring the importance of patient selection in determining treatment outcomes.

Omalizumab, which has been a mainstay for the treatment of allergic asthma with an IgE component for over a decade, is recommended for patients with perennial allergen sensitization and elevated IgE levels. Omalizumab trials have demonstrated a 25–50% reduction in exacerbations, with greater benefits observed in patients with high IgE levels and elevated eosinophil counts. The improvements in FEV_1_, however, tend to be modest. In contrast, dupilumab is not limited to allergic asthma; it can also benefit patients with non-atopic eosinophilic asthma, a phenotype that omalizumab does not effectively target. For patients who are allergic and eosinophilic, both omalizumab and dupilumab could be useful treatment options. A real-world study called ADVANTAGE compared these two agents in such patients and found that dupilumab was associated with significantly better outcomes, evident by a 44% lower exacerbation rate and 28% fewer OCS bursts over 1 year compared to omalizumab (Bleecker et al., 2024). While this was not a randomized trial, the outcomes suggest that, in practice, dupilumab’s broader mechanism may confer an efficacy edge in overlap phenotypes (e.g., allergic + eosinophilic) or in patients who had a suboptimal response to omalizumab. Another difference lies in the onset of action. While both drugs typically begin reducing exacerbations within a few weeks, some clinicians report that dupilumab can produce noticeable improvements in lung function as early as 2 weeks after initiation (Rhee et al., 2024). Omalizumab requires dosing based on IgE levels and weight, and high-IgE patients may need very high or split dosing, whereas dupilumab has fixed dosing that is not limited by IgE level, indicating convenience toward the latter in terms of dosing.

For the TSLP-targeting antibody, tezepelumab, which focuses on an epithelial cytokine upstream of the T2 inflammatory cascade, the drug approval of this agent was established in 2021 (Hoy, 2022). This drug has a unique ability to reduce exacerbations in asthmatic patients without the classic T2 biomarkers. In its pivotal trial, 50% exacerbation reduction was demonstrated even in low-eosinophil (<300) patients (Menzies-Gow et al., 2021), which neither dupilumab nor IL-5 antagonistic agents can claim at this low eosinophil count (Castro et al., 2018a; Akenroye et al., 2022). However, in T2-high inflammation patients, tezepelumab’s efficacy is comparable to that of dupilumab, with exacerbation reductions of 60–70%, yet no direct comparisons are currently available between those two agents. Dupilumab may offer more consistent improvements in lung function because its direct IL-13 blockade significantly influences airway physiology, whereas tezepelumab’s effect on FEV_1_ might be modest (Menzies-Gow et al., 2021). Tezepelumab may be a more suitable option in patients with mixed or non-T2 asthma, particularly those with low eosinophil counts and low IgE levels who continue to experience frequent exacerbations and are not candidates for other biologics. For patients clearly within the T2-high category, both dupilumab and tezepelumab are viable treatment options. In such cases, practical considerations such as injection frequency, cost, and drug accessibility may influence prescribing decisions. Notably, dupilumab is administered every 2 weeks, while tezepelumab is given every 4 weeks.

Generally, so far, for all asthma cases, biologic therapies have favorable safety profiles, with low rates of serious treatment-related adverse events in trials (Tan et al., 2019). Omalizumab carries a small risk of 0.1–0.2% of anaphylaxis, necessitating monitoring after injections and patient access to epinephrine, justifying the black-box warning included in the product label. Meanwhile, dupilumab has no such anaphylaxis signal in trials, evidenced by the very rare <0.1% anaphylaxis cases reported (Castro et al., 2018a). The anti-IL-5 therapies have occasional injection reactions, such as IV reslizumab, which had a reporting of a slight 0.2% anaphylaxis risk in trials, leading to monitoring recommendations (Cazzola et al., 2018; Li et al., 2021). Benralizumab can cause mild headaches or pharyngitis, but it notably causes rapid eosinopenia (Jackson et al., 2020). Additionally, the latter agent is generally well-tolerated but requires monitoring of parasitic or opportunistic infections. To reiterate in this context for dupilumab’s distinctive adverse effects, the transient blood eosinophilia can be detected in a minority of patients who are typically asymptomatic, and it usually resolves spontaneously, while conjunctivitis may surface in atopic dermatitis patients (Rabe et al., 2018) (Supplementary Figure S4). In asthma treatment with dupilumab, the incidence of conjunctivitis appears to be less of an issue in this population (Regeneron Pharmaceuticals IS, 2025), whereas it increases to 10% in eczema patients treated with dupilumab. The mechanism behind it may involve shifts in local immune balance in the eye, particularly due to IL-13’s role in maintaining ocular surface immunity (Deeks, 2019). Should conjunctivitis occur, it is usually manageable with ophthalmic therapy and does not necessitate stopping dupilumab (Agnihotri et al., 2019).

Another theoretical concern for IL-4/13 and IL-5 blockers is helminth infections, since T2 immunity considerably protects against parasitic infections. Clinical trial exclusion criteria ensure that patients have no helminth infections; therefore, cases of parasitic infection while on dupilumab or IL-5 agents are exceedingly rare (Tan et al., 2019). A review found no reported helminth infections in dupilumab asthma trials and similarly very low incidence in others, but the guidelines suggest treating any parasitic infection before initiating these biologics (Pera et al., 2023). Finally, tezepelumab’s safety concerns include a slightly higher incidence of nasopharyngitis and some instances of rash or injection-site reactions, which could take place, but there is no specific organ toxicity to be warranted in this regard (Hoy, 2022).

Altogether, dupilumab, with its unique dual targeting of the IL-4 and IL-13 pathways, exerts a broad anti-T2 inflammatory effect that partially overlaps with both anti-IgE and anti-IL-5 therapies. This allows dupilumab to demonstrate strong efficacy across a spectrum of asthma phenotypes, including overlapping allergic/eosinophilic asthma, purely eosinophilic asthma, and even some T2-low cases defined by lower eosinophil counts or other biomarkers such as FeNO. Dupilumab’s efficacy appears to be on par or even superior to that of other biologics when patients are appropriately selected based on T2 inflammation. Safety-wise, it avoids the anaphylaxis risk associated with agents such as omalizumab, offering a more reassuring profile for long-term use. Although its biweekly injection frequency can be considered moderate relative to omalizumab, which can be biweekly or monthly, or to benralizumab, which is every 8 weeks after induction, it is less appealing, but it remains within an acceptable range, especially when weighed against its clinical benefits. The choice of biologic therapy often hinges on the patient’s specific phenotype: omalizumab or dupilumab may be suitable for allergic asthma with normal eosinophils; anti-IL-5 or dupilumab is preferred in eosinophilic, non-allergic asthma; and in patients with comorbid conditions such as atopic dermatitis or nasal polyposis, dupilumab emerges as a particularly compelling option. For patients who do not clearly fit into the T2-high criteria, tezepelumab presents a valuable alternative. As head-to-head trials are currently lacking, real-world comparative studies, such as the ADVANTAGE study, are critical for refining therapeutic choices and guiding clinical decision-making (Bleecker et al., 2024). In comparative analysis with other biologics, dupilumab’s broader mechanism of action confers notable therapeutic advantages, especially in complex or overlapping asthma phenotypes characterized by concurrent allergic and eosinophilic features. Nonetheless, the decision to choose dupilumab over alternative biologics should remain closely aligned with individualized patient assessments, carefully considering specific phenotypic markers and clinical manifestations to optimize outcomes within a precision medicine framework.

## 9 Dupilumab in toxicological reports

Nonclinical toxicology studies of dupilumab did not reveal any significant safety concerns, which is consistent with its targeted mechanism and protein nature. Standard rodent toxicology models are not suitable for dupilumab because the antibody does not cross-react with the IL-4Rα of species such as mice or rats (Li et al., 2020; European Medicines Agency, 2017). Consequently, a surrogate antibody, termed REGN646 or REGN1033, that binds cynomolgus monkey IL-4Rα was used to evaluate toxicity in animal studies (Li et al., 2020; European Medicines Agency, 2017). Cynomolgus monkeys were given the surrogate IL-4Rα antibody at doses up to 100 mg/kg, administered weekly or biweekly for up to 6 months (European Medicines Agency, 2017). These surrogate doses are considered to represent very high exposures as they exceed human therapeutic levels by several-fold. The experimented monkeys tolerated dupilumab well, with no observed organ toxicity, clinical signs, or significant changes in body weight or food intake (European Medicines Agency, 2017). No macroscopic or microscopic pathological findings attributable to the drug were detected. Injection-site granulomatous inflammation occurred, consistent with high concentrations of human protein, but it was not dose-limiting. Additionally, there were no significant immunologic effects, including immune cell depletion or evidence of immunosuppression (European Medicines Agency, 2017).

One pharmacodynamic effect noted was a decrease in IgE levels in the treated monkeys, particularly in the shorter studies. This is consistent with IL-4Rα blockade, in which IL-4 is needed for IgE production, thus confirming that the surrogate was pharmacologically active. Importantly, treated monkeys were able to mount normal antibody responses to test antigens, indicating no overt immune deficiency from IL-4/IL-13 blockade (Li et al., 2020). The no-observed-adverse-effect level (NOAEL) was the highest dose tested of 100 mg/kg/week of anti–IL-4Rα antibody (Le et al., 2020), indicating a wide safety margin.

As a monoclonal antibody, dupilumab is not expected to have direct genotoxic DNA-damaging properties. In accordance with ICH S6 (R1) guidelines for biotechnology-derived pharmaceuticals, genotoxicity studies were not required as monoclonal antibodies do not interact with DNA or chromosomes (European Medicines Agency, 2017). Carcinogenicity studies, including long-term animal models, were also not conducted, which is standard practice for biologics such as dupilumab. Instead, a weight-of-evidence approach was employed. Preclinical data suggest that IL-4 and IL-13 signaling may promote tumor progression by skewing immune responses toward a Th2 phenotype, thereby suppressing anti-tumor Th1 immunity. Consequently, IL-4/IL-13 blockade may theoretically reduce tumor risk. Supporting this, chronic toxicity studies in monkeys showed no increase in pre-neoplastic lesions (European Medicines Agency, 2017). The European Medicines Agency’s assessment concluded that chronic IL-4Rα blockade is not associated with an increased risk of malignancy. IL-4/IL-13 blockade has been linked to reduced tumor progression in various tumor models (European Medicines Agency, 2017). Although long-term human data are still being collected, to date, no increased risk of malignancies has been observed in dupilumab clinical programs across different indications. Reproductive toxicology was evaluated in two species using surrogates (European Medicines Agency, 2017). In a fertility and early embryonic development study in a mouse model, a mouse analog of the dupilumab surrogate REGN1103 was administered subcutaneously at high doses (up to 200 mg/kg/week) to male and female mice prior to and during mating (USFaD, 2016). The results suggest no effects on mating behavior, fertility, or conception rates and no treatment-related changes in the uterus or ovaries of female mice or sperm parameters of male mice. Furthermore, no embryotoxic or teratogenic effects were noted in early gestation in the female mice (USFaD, 2016). For later-stage embryonic development, an enhanced pre- and postnatal developmental study was carried out in pregnant cynomolgus monkeys with the surrogate antibody of dupilumab. Pregnant monkeys were dosed throughout gestation at doses up to 100 mg/kg of the surrogate. Dupilumab, like other IgG antibodies, crosses the placenta, especially during the later phases of gestation, resulting in fetal exposure. The results showed no adverse effects on pregnancy, with no increase in miscarriages or stillbirths nor any abnormalities in the offspring due to the experimental dosing (Li et al., 2020; European Medicines Agency, 2017). Offspring born to treated mothers had no malformations and grew normally. There was a slight increase in infant eczema characterized by rash in the high-dose group of monkeys, hypothesized to be related to altering Th2 cytokine balance in neonates, but the report suggested that this was mild and transient. Overall, no evidence of teratogenicity or developmental toxicity was observed in preclinical studies (European Medicines Agency, 2017). While dupilumab has not been formally evaluated in clinical trials during pregnancy, case reports and registry data, primarily from women treated for atopic dermatitis, have not identified a clear teratogenic signal (Chopra et al., 2025). Thus, while IL-4 and IL-13 are not considered critical for fetal development, and the theoretical risk of their blockade appears limited, the current evidence remains insufficient to fully establish the safety of dupilumab use during pregnancy. Dupilumab should, therefore, be used in pregnant individuals with asthma only if the expected benefits outweigh potential risks, particularly when severe asthma cannot be controlled by other means. Further studies are needed to better understand the safety profile of dupilumab during pregnancy.

In another realm of toxicity, dupilumab was not tested for ecotoxicity in detail; as a protein, it is expected to be biodegradable in the environment. There were no notable off-target binding issues identified in a tissue cross-reactivity study using human tissues, with the notion that dupilumab bound only to the expected IL-4Rα-expressing cells. In addition, because dupilumab’s IL-4Rα target is mainly present on hematopoietic and some stromal cells, minimal binding is expected in non-immune organs, reducing the likelihood of off-target toxicity (USFaD, 2016).

Based on these reports, the toxicological profile of dupilumab appears reassuring. Preclinical animal studies using surrogate antibodies at high doses revealed no evidence of organ toxicity, carcinogenicity, or adverse effects on fertility or fetal development (Li et al., 2020; European Medicines Agency, 2017). These findings are further supported by clinical trial data, which similarly show no significant long-term safety concerns. The alignment between preclinical and clinical outcomes reinforces confidence in dupilumab’s therapeutic index. Toxicological assessments consistently highlight its targeted mechanism and minimal off-target effects, suggesting a favorable risk–benefit profile. These attributes support its use as a long-term therapy in chronic inflammatory conditions such as asthma, where sustained disease control must be balanced with patient safety.

## 10 Cost-effectiveness and pharmacoeconomics

The introduction of high-cost biologics such as dupilumab into asthma care raises important questions of cost-effectiveness and healthcare impact. Dupilumab’s list price is substantial in many regions, often ranging from approximately $30,000 to $40,000 per year, similar to other biologics used for asthma treatment (Wise, 2021; NIfHaC, 2021). Thus, pharmacoeconomic analyses from different countries have been conducted to determine whether the benefits of dupilumab justify its costs in various healthcare systems (Supplementary Table S1).

Several recent studies have assessed dupilumab’s cost-effectiveness as an add-on therapy for severe asthma, often comparing it to other biologics or standard care. Outcomes are usually measured in cost per quality-adjusted life year (QALY) gained (Supplementary Figure S5). Results have shown that dupilumab can be cost-effective in certain contexts, particularly when targeting patients who experience frequent exacerbations or are on maintenance OCSs.

For example, studies conducted between 2022 and 2024 in Colombia reported that in both asthmatic children and adults, dupilumab showed modest QALY gains in cost-effectiveness analyses. However, the incremental cost-effectiveness ratios (ICERs) exceeded the local willingness-to-pay (WTP) threshold of $19,000 per QALY (Ali et al., 2024; Buendía and Patiño, 2022; Antonio Buendía and Patiño, 2022) (Supplementary Table S1). Meanwhile, compared to other biologics, such as mepolizumab, benralizumab, and high-dose (450–600 mg) omalizumab, dupilumab has been found to be more effective and less costly. This was due to dupilumab’s efficacy in reducing expensive exacerbations and the high cost of those comparators in the local context. However, compared to standard-dose omalizumab of 300 mg, dupilumab was not cost-saving, and it produced more QALYs but at a higher cost, yielding an ICER of approximately $200,000, which is above the WTP threshold in this case (Ali et al., 2024). Therefore, it can be concluded from the Colombian reports that dupilumab is strongly dominant over IL-5 biologics and high-dose omalizumab, but it is not cost-effective compared to the lowest dose of omalizumab (Ali et al., 2024).

On another economic front, Japanese cost-effectiveness details on dupilumab were relatively encouraging (Supplementary Table S1). Dupilumab was slightly more effective than benralizumab and also less costly, making it the more cost-effective option in this comparison (Tohda et al., 2022). Compared to mepolizumab, dupilumab showed substantially greater clinical efficacy and only a modest increase in cost, resulting in an ICER of $9,190, which is well below the local WTP threshold of $45,000 per QALY, and therefore, it is considered cost-effective. In contrast, while dupilumab was also more effective than omalizumab, its cost was significantly higher, yielding an ICER of $98,203, which exceeds Japan’s WTP threshold. As a result, dupilumab was not deemed cost-effective relative to omalizumab in that comparative study (Tohda et al., 2022).

In South Korea, another cost-effectiveness study in 2024 evaluated dupilumab as add-on therapy in uncontrolled severe asthma from the Korean payers’ perspective (Oh et al., 2024). Using the local cost analysis and efficacy from that subpopulation trial data, they found that dupilumab added to standard care increased lifetime costs to approximately $113k relative to the $30k for standard care alone but also resulted in more than a twofold increase in QALYs (8.03 vs. 3.93), yielding a net gain of +4.10 (Oh et al., 2024). The resulting ICER was approximately $20,314 (Oh et al., 2024), which falls well below South Korea’s WTP of $27,000. Sensitivity analyses indicated a high probability of 87% that dupilumab is cost-effective at that threshold (Oh et al., 2024). Therefore, in South Korea, dupilumab was deemed to be a cost-effective option for severe asthma, especially considering the significant QALY gains, perhaps due to the drug’s increased benefit (Oh et al., 2024).

In the UK, it was found that in OCS-dependent severe asthma, dupilumab might be cost-effective due to a large reduction in corticosteroid-related morbidity costs (Wise, 2021; NIfHaC, 2021; Domingo et al., 2023). In 2022, national health technology assessments started evaluating dupilumab as part of the UK’s NICE study (NIfHaC, 2021). In this instance, NICE accepted an ICER just under the $36,400 threshold, supporting the cost-effectiveness of dupilumab. Thus, dupilumab is approved in this European country for patients with severe eosinophilic or high FeNO/T2 asthma who are not controlled with high-dose corticosteroids plus another controller and for those with comorbidities such as nasal polyps due to the drug’s increased benefit.

In the United States, cost-effectiveness results are sensitive to drug prices and the chosen comparator. In 2018, an analysis by the Institute for Clinical and Economic Review modeled new asthma pharmacoeconomics for biologic agents with suggestions for the US price list (Tice et al., 2018). The analysis suggests that dupilumab exceeded the conventional cost-effectiveness thresholds by >$150,000/QALY, making it not cost-effective compared to the standard care. Meanwhile, price discounts or targeting only the highest-risk patients, such as OCS-dependent patients, were deemed to be valuable and can improve the cost-effectiveness of this medication. A scenario analysis indicated that if the WTP threshold is increased to $150,000 per QALY, which is sometimes considered for severe diseases, dupilumab can be cost-effective compared to omalizumab (Yong et al., 2023). This implies that payers might negotiate pricing or restrict dupilumab use to patients with more severe disease in order to achieve better cost-effectiveness compared to other biologics such as omalizumab.

Thus, collectively, the cost-effectiveness of dupilumab varies significantly across countries due to differences in healthcare costs, payer perspectives, and WTP thresholds (Supplementary Figure S5). Dupilumab demonstrates favorable cost-effectiveness in some countries (e.g., Japan, South Korea, and the UK), but in others, such as Colombia and the US, it is not cost-effective at standard prices.

Pharmacoeconomic benefits beyond direct health outcomes include reductions in indirect costs, such as fewer missed work/school days due to asthma attacks, which translate into improved productivity. Severe asthma exacerbations and corticosteroid side effects carry a significant economic burden (Tice et al., 2018). Thus, by preventing these, dupilumab can offset some of its drug acquisition cost. For example, each hospitalization avoided saves thousands of dollars. Patients who can taper off maintenance prednisone may avoid costly long-term complications such as diabetes and fractures down the line. Cost-effectiveness models often account for these factors, which tend to favor dupilumab in subgroups with frequent exacerbations or steroid use.

Trading on budget impact analyses, we consider what adopting dupilumab broadly would mean for a healthcare system. Due to its high unit cost, if used widely in all eligible severe asthmatics, the immediate budget impact would be substantial. However, only a small fraction of all asthmatics qualifies for dupilumab, considering that 5–10% of the total cases are severe, including those with T2 biomarkers. In practice, utilization is also gated by prior authorization criteria, such as requiring failure of other biologics or the presence of certain eosinophil counts. Thus, the real-world budget impact has been manageable and in line with that of other biologics. Many payers treat asthma biologics as a class and often implement step-therapy protocols—for example requiring the use of anti-IL-5 agents first if eosinophils are >300 or omalizumab first in allergic patients—followed by dupilumab if those options fail or if the patient has multiple qualifying indications. Consequently, over time, competition among biologics could help drive costs down.

In summary, while dupilumab is costly, it may be a cost-effective option for patients with severe asthma, particularly for those with frequent exacerbations or chronic OCS use, where it provides substantial clinical benefit. Health–economic evaluations across multiple countries generally support its value in high-need subgroups, with ICERs often within acceptable thresholds when appropriate comparators are used (Ali et al., 2024; Oh et al., 2024). However, its cost-effectiveness is context-dependent and is influenced by healthcare system pricing, diagnostic access, and patient selection. In systems with strict prescribing criteria based on biomarkers and phenotypes, dupilumab demonstrates greater economic viability. Although its clinical benefits are well-established, affordability and reimbursement challenges persist in resource-limited settings. Targeting dupilumab to patients most likely to benefit is essential for ensuring both clinical effectiveness and cost sustainability. As real-world and long-term data continue to emerge, dupilumab may represent a valuable investment in precision asthma care, particularly in reducing systemic steroid dependence and improving quality of life.

## 11 Conclusion

Dupilumab has emerged as a first-in-class, highly effective biologic therapy for moderate-to-severe asthma and other T2 inflammatory diseases. By targeting both IL-4 and IL-13, it interrupts a key pathway that drives inflammation in the airways, skin, and mucosal tissues. In asthma specifically, this dual blockade addresses a central mechanism of disease, resulting in fewer exacerbations, improved lung function, better symptom control, and reduced reliance on corticosteroids. Its benefits extend across pediatric and adult populations with severe eosinophilic or corticosteroid-dependent asthma, which are consistently demonstrated through robust phase IIb and phase III clinical trials and real-world studies. As a result, dupilumab is now incorporated into major international guidelines as a recommended treatment option for T2-high asthma.

Beyond asthma, dupilumab’s broad anti-inflammatory action has led to its approval for multiple T2 inflammatory conditions, including atopic dermatitis, chronic rhinosinusitis with nasal polyps, prurigo nodularis, and eosinophilic esophagitis. In each case, its clinical use has been supported by rigorous trials and a favorable safety record, with adverse events being typically mild and rarely treatment-limiting. Unlike traditional immunosuppressants, dupilumab offers targeted precision therapy without broadly dampening the immune system, making its long-term use feasible and safe for many patients.

From a pharmacological and therapeutic standpoint, dupilumab represents a milestone in precision medicine, allowing treatment methods to be tailored based on a patient’s specific inflammatory profile. Emerging pharmacoeconomic data further suggest that when used in appropriately selected patients, dupilumab’s high drug cost may be offset by reduced emergency visits, fewer hospitalizations, and improved daily functioning and productivity. For many individuals who have long struggled with uncontrolled symptoms despite conventional treatments, dupilumab has brought meaningful relief, improved quality of life, and a chance to change the course of their disease.

Looking ahead, ongoing research is expanding dupilumab’s role, including studies in younger children and the investigation of additional biomarkers to further refine patient selection. As healthcare systems adapt and clinicians gain more experience with biologics, dupilumab is poised to remain a cornerstone in the management of severe asthma and related conditions—signaling a shift toward personalized, mechanism-based therapies that offer real hope to patients facing chronic, hard-to-treat inflammatory diseases.
