# Supplementary material for: Exploring dupilumab for asthma: from mechanistic insights to clinical outcomes, safety, and cost-effectiveness
Source: Front Pharmacol. 2025 Aug 6;16:1631321. doi: 10.3389/fphar.2025.1631321 (PMC12364877; doi:10.3389/fphar.2025.1631321)
Supplement: Supplementary file 1 [file Supplementaryfile1.docx]

Supplementary Material (Table)

**International Cost-Effectiveness Assessments of Dupilumab in Asthma Management Table 1.**

| Country | Population | Comparator(s) | QALYs (Dupilumab vs Comparator) | ICER (USD/QALY) | WTP | Cost-effectiveness | References |
| --- | --- | --- | --- | --- | --- | --- | --- |
| Colombia | Adolescents & adults with severe eosinophilic asthma | Standard therapy | ↑ QALYs | $50,160 | $19,000/QALY | ✗ | Antonio et al., 2022 |
|  | Children (6–11 y) with severe eosinophilic asthma | Standard therapy | ↑ QALYs | $24,660 | $19,000/QALY | ✗ | Buendía and Patiño, 2022 |
|  | Adults with severe uncontrolled asthma | Mepolizumab, Benralizumab, High-dose Omalizumab (450–600 mg) | ↑ QALYs, ↓ Cost | Dominant | $19,000/QALY | ✓ | Ali et al., 2024 |
|  | \| Adults with severe uncontrolled asthma \| \| --- \|  \|  \| \| --- \| | Omalizumab 300 mg | ↑ QALYs | $200,653 | $19,000/QALY | ✗ | Ali et al., 2024 |
| Japan | Patients ≥12 years with oral corticosteroid-dependent uncontrolled severe asthma | Benralizumab | ↑ QALYs, ↓ Cost | Dominant | $45,455/QALY | ✓ | Tohda et al., 2022 |
|  | Patients ≥12 years with oral corticosteroid-dependent uncontrolled severe asthma | Mepolizumab | ↑ QALYs, ↑ Cost | $9,190 | $45,455/QALY | ✓ | Tohda et al., 2022 |
|  | Patients ≥12 years with oral corticosteroid-dependent uncontrolled severe asthma | Omalizumab | ↑ QALYs, ↑ Cost | $98,203 | $45,455/QALY | ✗ | Tohda et al., 2022 |
| South Korea | Adolescents & adults (≥12 years) with uncontrolled severe asthma | Standard therapy | ↑ QALYs | $20,325 | $26,718/QALY | ✓ | Oh et al., 2024 |
| UK | Patients ≥12 years with severe asthma and type 2 inflammation | Standard therapy | ↑ QALYs | $36,000 | $36,400/QALY | ✓ | NIfHaC, 2021 |
| USA | Patients with moderate-to-severe uncontrolled asthma | Standard therapy | ↑ QALYs | $218,000 | $150,000/QALY | ✗ | Tice et al., 2018 |

✗ Not cost-effective

✓ Cost-effective

The table outlines international cost-effectiveness evaluations of dupilumab for asthma across various countries. It summarizes the incremental health benefits (measured in quality-adjusted life years, QALYs), associated incremental cost-effectiveness ratios (ICERs), and conclusions regarding cost-effectiveness relative to local willingness-to-pay (WTP). Note that the estimated currency values in this table were all converted to USD equivalent based on the exchange rate corresponding to the referenced study year.

"Dominant" refers to cases where Dupilumab was both more effective (yielded more QALYs) and less costly than the comparator.

WTP refers to the willingness-to-pay threshold, which is the maximum amount a healthcare system is willing to pay per QALY gained. If the ICER falls below this threshold, the treatment is considered cost-effective (✓); if above, it is not (✗).

All cost values have been converted to 2022–2024 USD equivalents based on exchange rates reported in the respective studies.
