# Supplementary material for: Exploring dupilumab for asthma: from mechanistic insights to clinical outcomes, safety, and cost-effectiveness
Source: Front Pharmacol. 2025 Aug 6;16:1631321. doi: 10.3389/fphar.2025.1631321 (PMC12364877; doi:10.3389/fphar.2025.1631321)
Supplement: Supplementary file 2 [file Supplementaryfile2.docx]

Supplementary Material (figures)


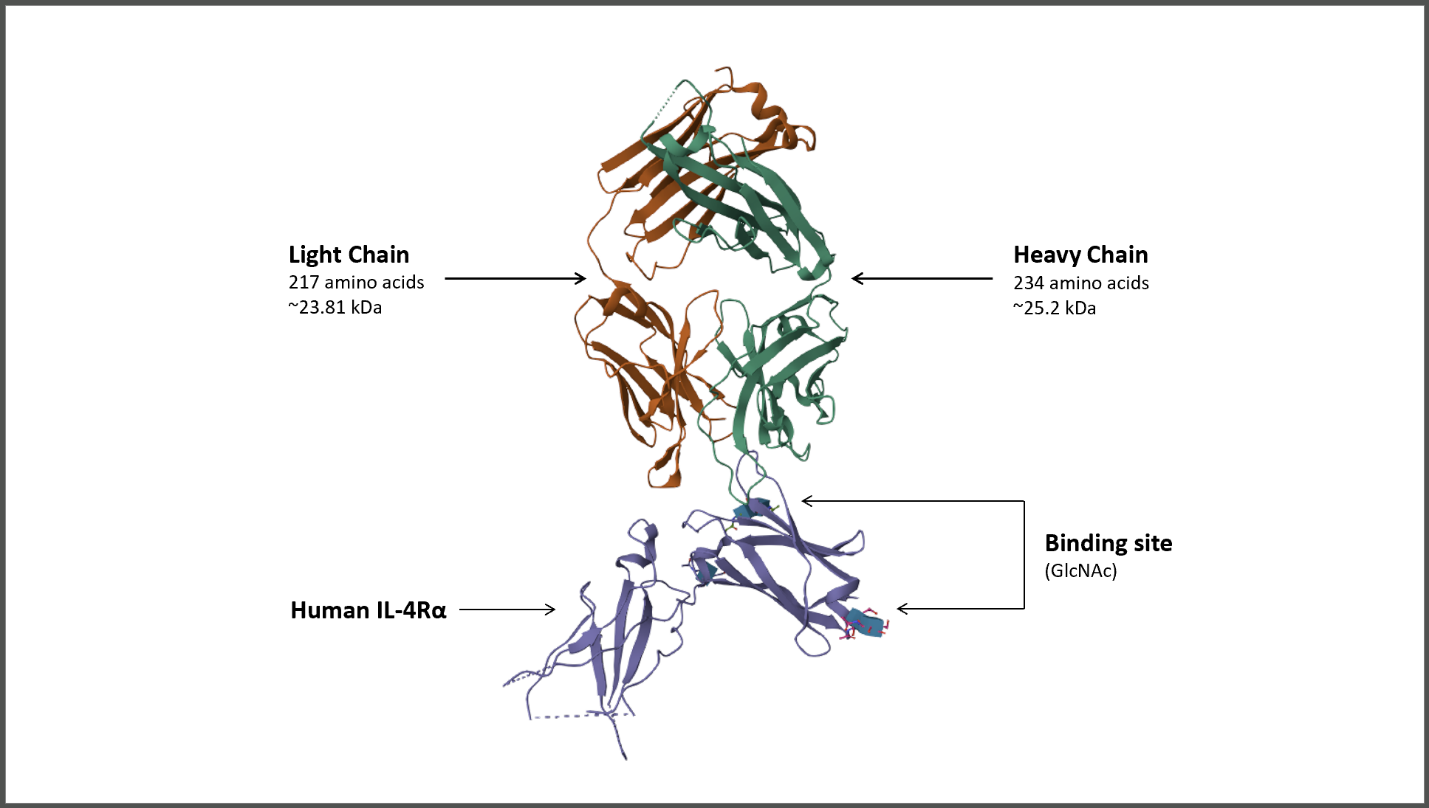


**Crystal Structure and Molecular Composition of dupilumab Figure 1.** A 3D structural representation of the Fab portion of Dupilumab bound to the human IL-4 receptor alpha subunit (IL-4Rα). The light chain, consisting of 217 amino acids (~23.81 kDa), is shown in brown, while the heavy chain, consisting of 234 amino acids (~25.2 kDa), is shown in green. The receptor component, IL-4Rα, is depicted in purple. The binding site, indicated by the presence of GlcNAc (N-acetylglucosamine), highlights the interaction site where Dupilumab inhibits IL-4 and IL-13 signaling.

The complete molecular weight of Dupilumab (147 kDa) includes the entire antibody structure, comprising two Fab fragments (heavy and light chains) and the Fc region (consisting of heavy chain constant domains). The Fc region (not shown in the figure) significantly contributes to the overall weight, adding approximately 50 kDa. Figure adapted and modified from (Regeneron Pharmaceuticals IS, 2025; McCann et al., 2024).


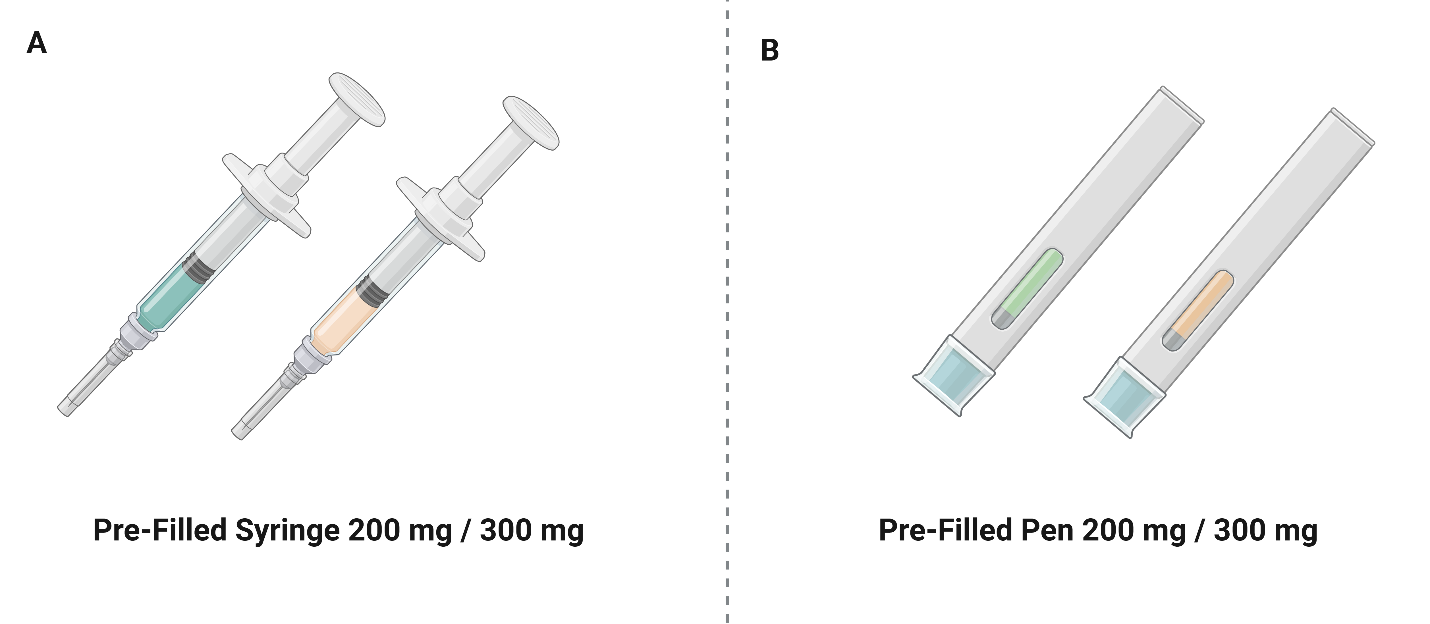


**Commercial Formulations of Dupilumab Figure 2.** (A) Pre-filled syringes containing either 200 mg or 300 mg of dupilumab in solution, typically used for subcutaneous injections. (B) Pre-filled pens containing either 200 mg or 300 mg of dupilumab in solution, designed for easier self-administration by patients. Authors fully created figure 2 via scientific illustration software; BioRender.com.

**
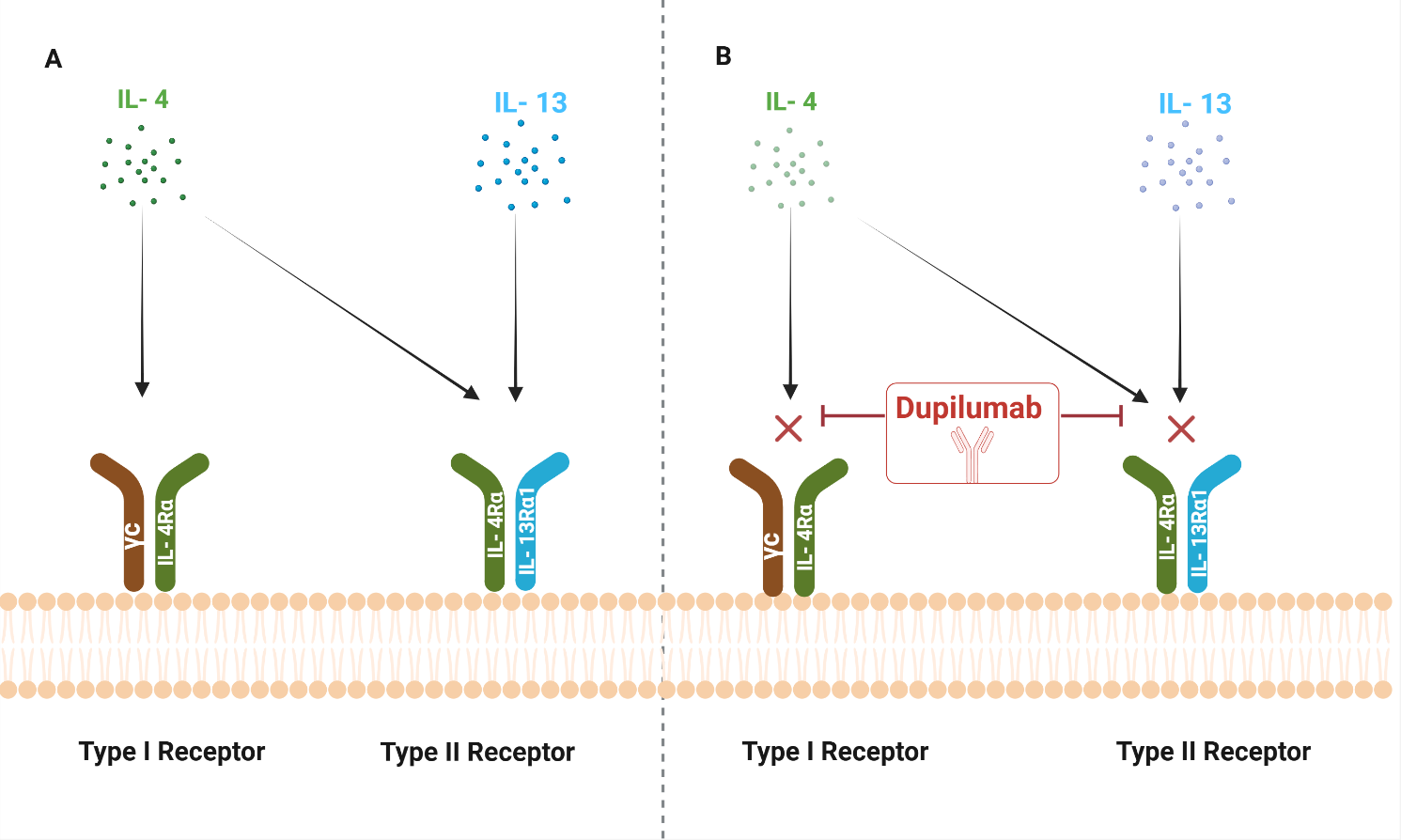
**

**Mechanism of Action of Dupilumab on IL-4 and IL-13 Signaling Pathways Figure 3.** (A) In the absence of dupilumab, IL-4 and IL-13 cytokines signal through Type I (IL-4Rα/γc) and Type II (IL-4Rα/IL-13Rα1) receptor complexes, respectively, triggering downstream pro-inflammatory responses characteristic of type 2 inflammation in asthma. (B) Dupilumab, a monoclonal antibody targeting IL-4Rα, blocks the shared receptor subunit, thereby inhibiting both IL-4 and IL-13 signaling. This prevents receptor activation and downstream effects such as IgE production, eosinophil recruitment, and mucus hypersecretion. Authors fully created figure 3 via scientific illustration software; BioRender.com.


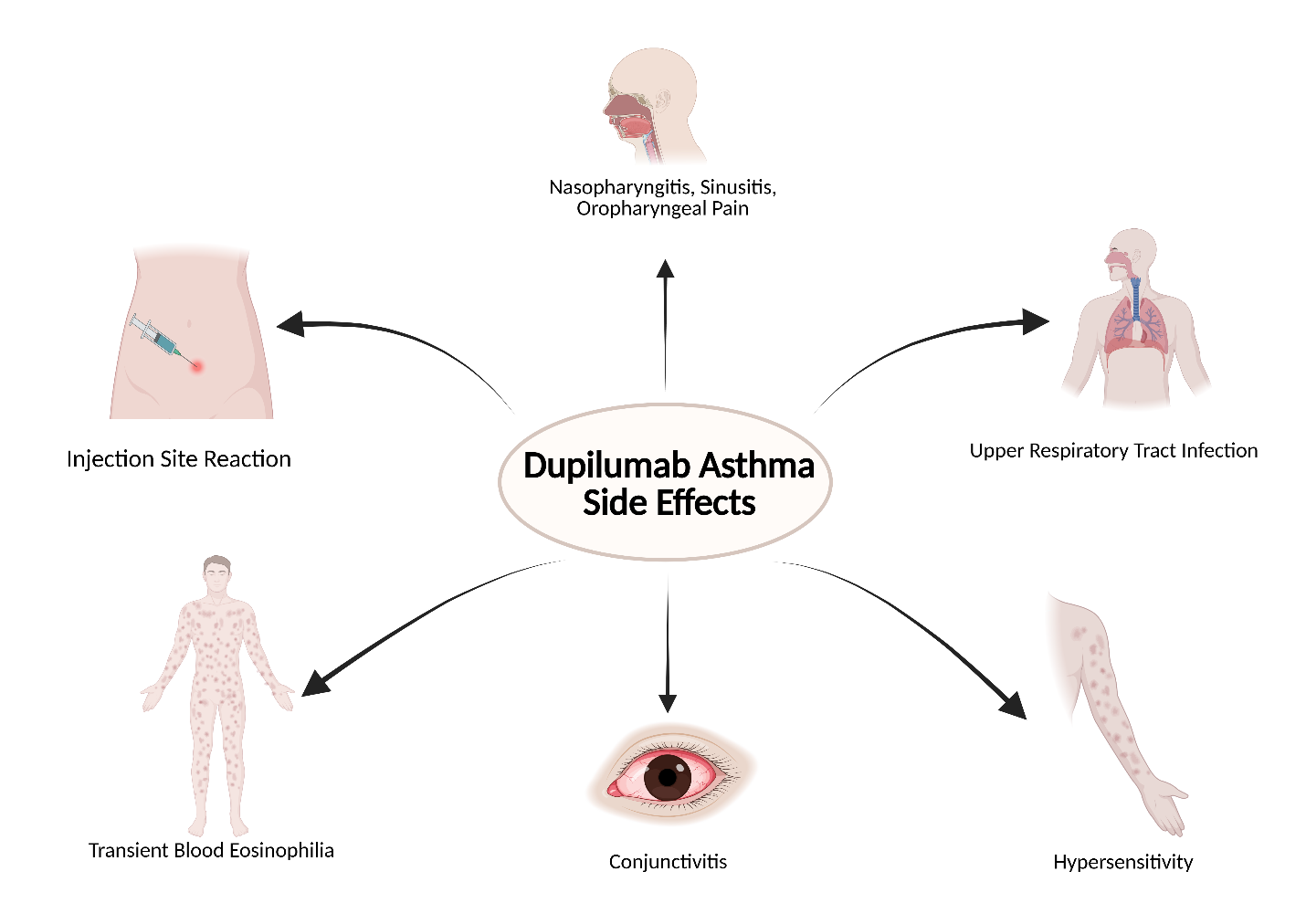


**Summary of Side Effects Observed in Clinical Trials of Dupilumab for Asthma Figure 4.** Common adverse events associated with dupilumab include local injection site reactions, systemic effects like headache and transient blood eosinophilia, and respiratory-related symptoms including nasopharyngitis, sinusitis, and oropharyngeal pain. Conjunctivitis has been reported more frequently in patients with atopic dermatitis but is less common in asthma. Hypersensitivity reactions, though rare, may occur. Authors fully created figure 4 via scientific illustration software; BioRender.com.


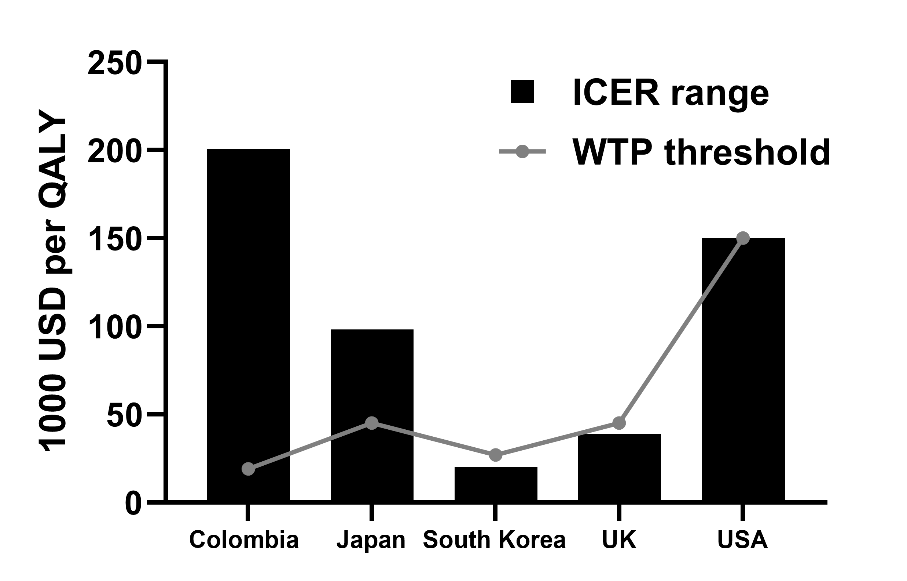


**Cost-Effectiveness of Dupilumab in Severe Asthma Across Different Countries Figure 5.** Dupilumab demonstrates favorable cost-effectiveness in some countries (South Korea, UK, Japan when compared to certain biologics) but in others, such as Colombia and the US, and in Japan versus omalizumab, it is not cost-effective at standard prices.

ICER: The incremental cost-effectiveness ratio, QALY: Quality-Adjusted Life Year. WTP threshold represents the maximum amount a healthcare system, payer, or society is willing to pay for one additional QALY gained from a treatment. Note that the estimated currency values in this figure were all converted to USD equivalent based on the exchange rate corresponding the respective country report.
